# An improved database of coastal flooding in the United Kingdom from 1915 to 2016

**DOI:** 10.1038/sdata.2017.100

**Published:** 2017-08-01

**Authors:** Ivan D. Haigh, Ozgun Ozsoy, Matthew P. Wadey, Robert J. Nicholls, Shari L. Gallop, Thomas Wahl, Jennifer M. Brown

**Affiliations:** 1 Ocean and Earth Science, University of Southampton, National Oceanography Centre Southampton, University of Southampton, Waterfront Campus, European Way, Southampton SO14 3ZH, UK; 2 Engineering and the Environment, University of Southampton, Highfield, Southampton SO17 1BJ, UK; 3 Eastern Solent Coastal Partnership, Havant Borough Council, Southmoor Depot & Offices, 2 Penner Road, Havant PO9 1QH, UK; 4 Department of Environmental Sciences, Macquarie University, Sydney, North Ryde 2109, Australia; 5 Department of Civil, Environmental, and Construction Engineering and Sustainable Coastal Systems Cluster, University of Central Florida, 12800 Pegasus Drive, Suite 211, Orlando, Florida 32816-2450, USA; 6 National Oceanography Centre, Joseph Proudman Building, 6 Brownlow Street, Liverpool L3 5DA, UK

**Keywords:** Physical oceanography, Projection and prediction, Natural hazards

## Abstract

Coastal flooding caused by extreme sea levels can produce devastating and wide-ranging consequences. The ‘SurgeWatch’ v1.0 database systematically documents and assesses the consequences of historical coastal flood events around the UK. The original database was inevitably biased due to the inconsistent spatial and temporal coverage of sea-level observations utilised. Therefore, we present an improved version integrating a variety of ‘soft’ data such as journal papers, newspapers, weather reports, and social media. SurgeWatch2.0 identifies 329 coastal flooding events from 1915 to 2016, a more than fivefold increase compared to the 59 events in v1.0. Moreover, each flood event is now ranked using a multi-level categorisation based on inundation, transport disruption, costs, and fatalities: from 1 (Nuisance) to 6 (Disaster). For the 53 most severe events ranked Category 3 and above, an accompanying event description based upon the Source-Pathway-Receptor-Consequence framework was produced. Thus, SurgeWatch v2.0 provides the most comprehensive and coherent historical record of UK coastal flooding. It is designed to be a resource for research, planning, management and education.

## Background & Summary

Of all natural hazards, coastal flooding due to extreme sea levels has caused some of the worst human and financial losses worldwide^1^. Major events since 1900 include the Galveston, Texas hurricane in 1900 (ref. 2), Cyclone Bhola in the Bay of Bengal in 1970 (ref. 3), Hurricane Katrina in New Orleans in 2005 (ref. 4) and Typhoon Haiyan in the Philippines in 2013 (ref. 5). Chronologies compiled for flood-prone regions^6^ indicate that storm surges continue to cause widespread devastation^7,8^, despite advances in risk management and technology (e.g., flood defences, forecasting and warning). Coastal flooding is a growing threat due to climate-induced sea-level rise^8^, land subsidence^9,10^ and rapid population and economic growth in flood-exposed areas^11,12^.

In the UK it has been estimated that 4 million people and £150 billion of assets are threatened by coastal flooding^13^. Coastal flooding is rated as the second highest risk of civil emergency in the UK, after pandemic influenza^14^. Coastal flooding, combined with fluvial flooding, is responsible for at least £0.25bn in annual economic damages^15^. The UK has a long history of severe coastal flooding. Historic accounts suggest that large numbers of people (of order 10^5^ per event) were drowned on the east coast in 1099, 1421 and 1446, but with large uncertainty^16^. In 1607, floods around the Bristol Channel killed around 2,000 people^17^, and coastal flooding caused by the ‘Great Storm’ of 1703 ‘completely washed away’ the lower streets of Brighton on the south coast^18,19^. In the last century, major events include the 1928 flood which drowned 14 people in central London^20^, and the ‘Big Flood’ of 31st January–1st February 1953 that devastated the east coast, with 307 deaths and 24,000 evacuees^21,22^. The 1953 event was pivotal in shaping the current state of UK flood risk management, and was a major incentive for scientific research and improved forecasts, warnings, and sea defences^23,24,25,26^. Recently, the winter of 2013–2014 saw severe storms and extreme sea levels, resulting in widespread and prolonged coastal flooding^27,28,29,30,31,32^.

Motivated by the absence of a national framework for documenting coastal floods and their impacts, we^32^ developed a coastal flood database and online tool called ‘SurgeWatch’ (v1.0; Data Citation 1) the first-step to provide a systematic record of coastal flood events around the UK from 1915 to present. We subsequently used it to improve understanding of the spatial and temporal characteristics of extreme sea-level events around the UK^31^. However, as we previously acknowledged^32^, SurgeWatch1.0 had two key limitations: (1) historical events were omitted or under-represented due to the incomplete coverage of tide gauge records, which are sparse pre-1980s and especially prior to the mid-1960s; and (2) events were ranked using the maximum sea level return period, but the extent and severity of coastal flooding is more complex than this owing to other variables (e.g., waves and defences).

This paper presents SurgeWatch2.0, which encompasses significant updates to address these two issues. First, we systematically reviewed a wide range of ‘soft’ data sources which document instances of UK flooding and storms. This resulted in the identification of 329 coastal flood events from 1915 to the end of 2016, which is a fivefold increase on the 59 coastal flood events identified in the original database. Second, events were classified based on impacts using a multi-level categorisation: ranging from 1 (Nuisance) to 6 (Disasters). In addition, we replaced the existing template for event descriptions with an enhanced systematic commentary based on the Source-Pathway-Receptor-Consequence (SPRC) model—the most widely accepted conceptual model representing all interacting elements of the coastal floodplain system^33,34^. SurgeWatch2.0 continues to be available online in an enhanced user-friendly website (www.surgewatch.org).

The systematic assessment of the coastal flood events and their consequences is foundational for developing a thorough understanding of the flood system, and for making better decisions regarding the allocation of public resources for flood defences and management. Therefore, SurgeWatch2.0 will be useful for both scientific and practical applications, and of interest to a wide range of audiences. We hope that similar datasets will be compiled for other countries/regions, following the framework we have developed here.

## Methods

Creating SurgeWatch2.0 involved three main stages, explained in detail below.

### Stage 1: Review of ‘soft’ data sources

To create SurgeWatch1.0, we first used sea-level records from the National Tide Gauge Network (Data Citation 2) and extracted all extreme sea-level events that reached or exceeded the 1 in 5 year return level. Across the 40 tide gauge sites we analysed, 310 high waters reached or exceeded this threshold, resulting from 96 distinct storms. We used the dates of these 96 events as a chronological base from which to investigate whether historical documentation exists for a concurrent coastal flood. We found evidence of flooding for 59 out of the 96 storm events. Given that our initial focus was on dates extracted from sea-level records, it is almost inevitable the original database missed several events due to incomplete coverage of tide gauge records which are sparse pre-1980s and especially prior to the mid-1960s. This method was also inherently liable to omit flood events that arose from when sea levels were lower than the 1 in 5 year threshold chosen, and/or when coastal flooding was predominantly caused by factors other than storm surges, such as large waves and tide-locking of rivers. Therefore, to developed SurgeWatch2.0 we compiled a more comprehensive record of historical coastal flood events, by undertaking a detailed review of the known available ‘soft’ data sources that document instances of UK storms and floods.

We closely reviewed nine main sources, namely: Lamb^35^, Davison *et al.*^36^, Hickey^37^, Zong and Tooley^38^, Eden^39^, Ruocco *et al.*^40^, Kundewicz *et al.*^41^, Stevens *et al.*^12^, and Haigh *et al.*^32^. A brief description for each source, the periods they cover, and the sources they draw upon, is given in Table 1 (available online only). There is a variable number of coastal flood events documented in each source, in addition to disparities in the level of detail provided; some list only the date of the event, with no further detail. Three of the sources provide only local to regional scale flood histories (Davison *et al.*^36^—Hampshire and Isle of Wight; Hickey^37^—Scotland; Ruocco *et al.*^40^—the Solent), whilst all other sources provide a national perspective. These nine sources are not exclusively concerned with coastal flooding, and some focus on other aspects of extreme storms and other weather-related hazards such as hail and tornadoes (Lamb^35^; Davidson *et al.*^36^; Eden^39^), for which we recorded only the events where coastal flooding was explicitly mentioned. Stevens *et al.*^12^ documented instances of both fluvial and coastal flooding mentioned in the monthly weather and hydrological reports provided by the Met Office (http://www.metoffice.gov.uk/learning/library/archive-hidden-treasures/monthly-weather-report) and Centre for Ecology and Hydrology (http://nrfa.ceh.ac.uk/monthly-hydrological-summary-uk). Kundewicz *et al.*^41^ recorded large fluvial and coastal floods globally. In these instances, we recorded only the coastal flooding events. To our knowledge, these sources provide the most relevant and up-to-date chronologies available in the UK that are concerned with storms or coastal flooding, and collectively provide a sufficiently detailed overview of historic events. Although several of the nine sources recorded events pre-1915, we focus here on the 102-year period from 1915 onwards, as a first-step to make the task manageable. Most sources provide coverage since 1915.

The nine key sources cite many other documents, such as periodicals, newspaper articles, flood and extreme weather chronologies, monthly weather and hydrological reports, journal papers, professional reports, and other online sources (e.g., blogs, social media). Where possible, we verified these original sources and obtained further information. We also used focused analysis of contemporary (i.e., at the time of the event) newspaper articles in The Times Digital Archive (http://gale.cengage.co.uk/times.aspx/) and the National Library of Australia (http://trove.nla.gov.au/) to obtain additional information for individual events. However, we did not undertake extensive searches of these archives beyond the dates of events identified by the nine main sources.

For each event identified, we recorded the: date of the event; country or region affected; specific locations mentioned in the source; and the specific source(s) listing that event. We added additional events we were aware of from other sources. As discussed in more detail in the Technical Validation section, we then identified and combined duplicate events where: (1) different sources provide separate reports for an event each dated over different consecutive days; and (2) no specific day or month is reported and there were other events within the same month (year) which are likely to be the same event.

**Figure 1:**
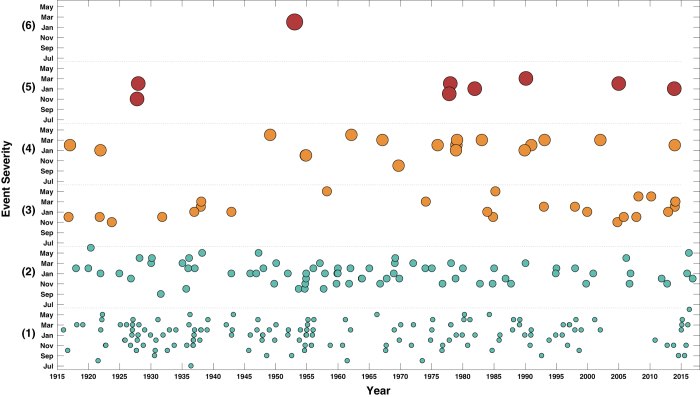
Ranking of each of the events in chronological order. .

In total, we identified 329 distinct coastal flood events from the start of 1915 to the end of 2016 (Table 2 (available online only) and Fig. 1). These are defined as events with a period of high sea levels and/or waves arising from a distinct storm, which were associated with coastal flooding. For several events only the month or year in which they occurred are reported. In some cases, as discussed in more detail in the Technical Validation section, we were able to identify the day or month from other sources or through our own analysis of meteorological and sea-level data. However, 43 events remain where we know the month of occurrence only, and nine for which we only know the year. We have included these in the database as more detailed information may come to light in the future.

For each of the 329 coastal flood events identified, we documented (described in detail in Stage 3) all information we could find about the event using: (1) journal papers; (2) publically available reports and newsletters by interested professional parties such as the EA, Meteorological Office, local councils and coastal groups; (3) journalistic reports/news websites; and (4) other online sources (e.g., blogs, social media). The level of information available for each event varies greatly, but as an absolute minimum we identified a date and a location impacted.

### Stage 2: Categorising events based on the severity of their consequences

In SurgeWatch1.0, we ranked coastal flooding events by the estimated maximum sea level return period for each event, using tide gauge data. As a result of poor coverage, several events were ranked lower than they should be in the original database. This is because, while we have tide gauge data at some sites for these events, tide gauges were not necessarily operational at the time along the stretches of the coastline where the sea levels were likely to have been most extreme. For example, the 31 January–1 February 1953 event was ranked 10th in the original database, but we know from examining the event in detail^30^, and considering other information sources (Rossiter^42^ in particular), that it should be ranked highest, both in terms of maximum sea level return period and impact. Only four of the 40 tide gauges were operational at that time and the gauge closest to the location of the peak storm surge failed during the event, just prior to high water, and the next closest (Newlyn) was located too far away. In addition, the severity of a coastal flood is not linearly related to the maximum sea level return period, due to other important variables such as waves, the presence and state of defences, and population density. Thus, the aim of the second stage was to rank all events using a multi-level categorisation based on reported impacts of the flooding, which is independent of the storm or water level characteristics, to more accurately represent the severity of the events in terms of impacts.

We used an iterative process to devise a simplified multi-level categorisation to classify flood events based on the severity of their consequences. All flood events were classified (with a higher score representing higher consequence), as either: (1) Nuisance; (2) Minor; (3) Moderate; (4) Major; (5) Severe or (6) Disaster. We refined the classification so that the number of events in each category roughly reduced exponentially, as would be expected in reality. Comparatively, the Richter scale, used to quantify the size of an earthquake, varies on base-10 logarithmic scale. The criteria to define these six categories, are listed in Table 3, and were based on the information yielded from Stage 1. They closely reflect our understanding of the key impacts that delineate the severity of coastal floods, but are also inevitably influenced by the style of reporting and availability of information.

Category 1 events (Nuisance) are those with localised flooding, where roads, parks or quayside areas were flooded. Events where we were only able to identify the location(s) flooded and no specific consequences were also ranked 1. For Category 2 events (Minor) the inundation was typically more extensive and in most cases properties were flooded and/or there was disruption to services. Category 3 (Moderate) events involved flooding of larger number of properties, wider disruption to services and/or flooding of agricultural land. Category 4 (Major) and 5 (Severe) events involved more extensive flooding with significant damage to infrastructure and large economic damage costs. For an event to be ranked Category 5 there needed to be either loss of life due to drowning, or reliable evidence that defences and/or flood warnings, and a substantial institutional response to the event, prevented multiple fatalities. Category 6 events (Disaster) were reserved for large consequence events that are associated with multiple fatalities due to drowning. Direct flood-related fatalities caused by immediate physical trauma (primarily drowning) are linked to only six UK floods since 1915. Of the 329 events in the database, 185 were ranked Category 1, 91 were ranked Category 2, 26 Category 3, 18 Category 4, 8 Category 5, and only 1 (the 31st January–1st February 1953 event) was ranked Category 6.

### Stage 3: Improving event descriptions by incorporating the SPRC framework

**Figure 2:**
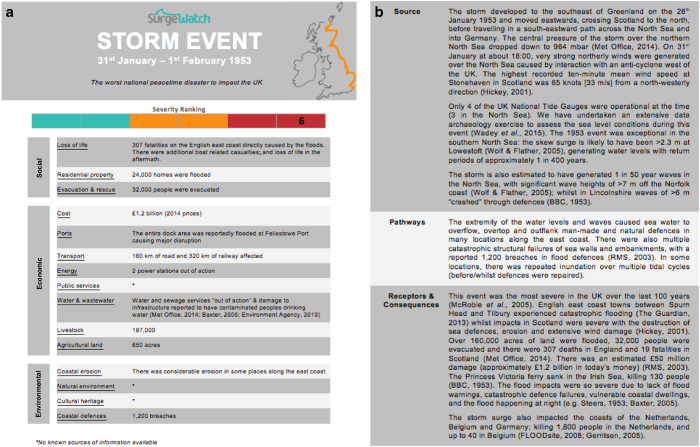
Example (31st January-1st February 1953) of the (**a**) first and (**b**) second pages of the commentaries for Category 3 and higher events.

In the third and final stage, we compiled a systematic commentary for each of the 329 events. As expected, there was often only limited information available for the lower ranked events. Hence, for each of the Category 1 events we provide a one or two-sentence summary of the reported consequences. The same was done for each of the Category 2 events, but with a longer paragraph describing the reported consequences. For the Category 3 events and higher, for which more information was available, we compiled a longer systematic commentary. Each of these includes, on the first page, the event date, a map indicating the approximate stretch of coast where flooding was reported, and a one sentence summary in the style of a news headline. This is followed by a color-coded graphic indicating the event ranking, with: Category 1 and 2 events in turquoise; Category 3 and 4 in orange; and Category 5 and 6 in dark red. A significant enhancement of the event commentaries in SurgeWatch2.0 is the addition of a table outlining 15 key parameters which highlight the types of consequence criteria associated with the events that were reported (3 social, 8 economic and 4 environmental). Examination of these parameters for the events was key to establishing and refining the criteria we used to define the six ranking categories, as described above. An example of the first page of a Category 3 or higher event commentary is shown in Fig. 2a. Each commentary includes on the second page a concise narrative of the event, an example of which is shown in Fig. 2b. In SurgeWatch1.0 this narrative contained, in three sections respectively, a description of: (1) the meteorological conditions; (2) the sea-level conditions experienced during the event; and (3) a succinct account of the recorded consequences to people and property. In hindsight, we reflect that it is more appropriate to reformat this narrative around the Source-Pathway-Receptor-Consequence (SPRC) model; and hence have done this for SurgeWatch2.0. Importantly, defining the SPRC elements of events in our database improves clarity and compatibility for integration of SurgeWatch2.0 with other flood analysis literature.

The Source describes the natural drivers of coastal flood events (i.e., tide, surge and waves, and the associated meteorological conditions). The Pathways component comprises descriptors (e.g., reporting or photos) of flood defence responses (e.g., erosion, breaching, overtopping, overflowing) and inundation (e.g., flood water surrounding or entering buildings). Receptors can be regarded as anything that can suffer damages from the event, and in our assessment, are regarded as people, property, infrastructure and the environment impacted by flooding. Finally, Consequences (defined as impacts upon people, property, the economy and the environment) is a key component of SurgeWatch2.0, as this is the most meaningful measure to determine the severity of a flood event in terms of affecting society (albeit one that is intrinsically linked with the sources, pathways and receptors).

In the revised narrative, the first section describes the source of the event (combining sections 1 and 2 in the SurgeWatch1.0 narrative). This is separated into three paragraphs: the first describing the meteorological conditions of the storm; the second outlines the still water level conditions; and the third the wave conditions observed during the event. These were characterised from information reported in the available sources and using the datasets and methods established for the original database. We provide only a brief overview of the datasets and methods that were used to characterize sources here; Haigh *et al.*^32^ provide a comprehensive account. We used global reanalysis^43^ data provided at 6-hourly intervals (Data Citation 3) to determine key meteorological features of the events (e.g., the storm track, atmospheric pressure and wind speeds and directions). We used records from the National Tide Gauge Network (Data Citation 2) to establish still water level conditions, including the astronomical tide and skew surge^44^ components, observed during each event. Sea level return periods were estimated using exceedance probabilities from an Environment Agency study^45,46^. Due to the limited spatial and temporal coverage provided by the tide gauge observations (which is most noticeable for the period pre-1960s), we could only document the peak sea-level height(s), astronomical tides and skew surge(s) for events which coincide with the data records available. For wave conditions, we referred to reported information where available.

In the revised narrative, the second section briefly describes the pathway of the flood, as reported in the available sources. For older events, information on pathways is often missing and we explicitly acknowledge this in the narrative. The third section briefly but systematically describes the receptor and consequences for the event, as reported in available sources. The first paragraph focuses on the consequences related specifically to the coastal flood. The second paragraph, where relevant, briefly describes impacts that relate to other aspects of the storm (i.e., wind damage) that are not directly relevant to coastal flooding. The third paragraph, if relevant, describes coastal flood impacts that occurred in neighbouring counties; which is only the case for the very large events.

Each commentary also includes (as in SurgeWatch1.0) a graphical representation of the storm track, mean sea level, pressure, and wind fields at the time of maximum high water. They also include figures of the return period and skew surge magnitudes at sites around the UK, and a table containing the available peak sea-level measurements for each event.

## Data Records

SurgeWatch2.0 is available to the public through an unrestricted repository available at the British Oceanographic Data Centre (BODC) portal (Data Citation 4), and remains formatted according to their international standards. It includes data available up to the end of 2016. The first file is a spread sheet (XLSX) containing the list of all 329 coastal flood events in the database categorised according to the severity scale that we devised. The second and third files are PDF documents containing the short commentaries for all Category 1 and 2 events. There are an additional 53 PDF files containing the longer event commentaries for events ranked Category 3 and higher. Two final CSV files contain: the digitised storm tracks for the 53 Category 3 and higher events; and the peak sea-level height(s), astronomical tides and skew surge(s) for events which coincide with the data records available. Each of these files is self-describing and is accompanied by extensive metadata.

The database remains freely available at the SurgeWatch website (http://www.surgewatch.org), along with interactive graphical presentations of event-specific sea level return periods and skew surges, a glossary of key terms, educational videos, and news articles. We have enhanced the design of the website. The home page now includes a navigable time-line by which users can scroll through the events in reversed chronological order. Where we can, we have added photos taken at the time of the event. The website has been designed to crowd source additional information. There is a facility for users to upload photos they may have of any event(s). Photos get moderated before showing up against that event.

## Technical Validation

SurgeWatch2.0 was developed by undertaking an extensive search of documentation from a variety of readily available ‘soft’ data sources. In addition, it uses freely available and easily accessible meteorological and sea-level datasets, which have undergone rigorous quality control and validation. We have built on our previous experience of compiling the original dataset and addressed key issues to create a significantly enhanced record of coastal flooding for the UK for the last 102 years. We now outline a number of methodological issues encountered in the process of compiling the updated database.

The first issue relates to the fact that the quantity and quality of the reporting of coastal flooding varied significantly. Unsurprisingly, the amount of documentation available on coastal flooding improves with time, and more recent events are reported in greater detail. Several notable events in the earlier portion of the record (e.g., the 6th–7th January 1928 flooding in central London) are, however, also well documented. The most useful source for information on consequences of coastal floods was newspaper articles. Newspaper articles generally focus on human dimensions of flooding, while peer-reviewed journal papers are more dependable, but tend to focus more on analysis of the sources and pathways of events. Most sources are secondary or tertiary, and hence we were not able to identify the original source in many cases. We attempted to compare information about each event between multiple sources to verify the validity of the facts (e.g., date and time, main areas affected and impacts). However, this was not always possible as some sources (e.g., Lamb^35^; Eden^39^) are not fully independent and were compiled using common (primary) sources such as contemporary newspaper articles. Therefore, an event mentioned in more than one source, and reportedly involving considerable flooding, may be exaggerated if these are secondary or tertiary sources and all are based on the same primary source alone.

We overcame some of the key issues relating to ambiguous dates and duplicate events, by systematically (and independently) examining meteorological and sea-level data. In some cases, it appeared likely that the sources were referring to the same event, but on different days. For example, Zong & Tooley^38^ reported flooding in Perth, Scotland and the wider region of Tayside on the 8th January 1920, whilst Hickey^37^ reports flooding in Dundee also within Tayside, on the 9th January 1920. It was not immediately clear whether these were two distinct coastal flood events, caused by two different storm systems in close succession, or the same event caused by one storm. For each case when different sources reported events on consecutive days, we systematically examined the meteorological reanalysis and sea-level data (Data Citation 2 and Data Citation 3) available at the time to determine whether these were the same event, which impacted the coast over two days, or two distinct events, arising from different storm systems. For transparency, we explicitly record that we combined events in the commentaries where relevant.

An important component of producing this enhanced dataset was to illustrate the severity of flood events according to their consequences. This was achieved by devising a multi-level categorisation using criteria based on the pathways, receptors, and consequences of flood events. Although the scale represents a simplification of flood impacts, it does provide a meaningful classification that reveals the variety of flood types. Inevitably there is subjectivity in the ranking system. Ideally, these categories would be based entirely on more transparent, quantitative criteria (e.g., damage costs, recovery time). However, the information we identified from the sources was largely qualitative. Consequently, some events were not easily classified, e.g., such as those that had a large spatial footprint (i.e., affecting one or more coastal sectors), but simultaneously did not produce severe consequences. The broad definition of the categories however, did facilitate classification. We used an iterative process to refine the categories and number of events in each. We tried to ensure that ranking of each event was based on the consequences that arise due to the coastal flood directly, as opposed to other variables relating to the storm, such as high winds. In the case of loss of life, it was sometimes difficult to distinguish this. Despite the limitations, the classification provides a useful indication of the variety of flood types over the last 102 years. We deliberately designed the ranking system to increase numerically with severity, so we could add a larger category (i.e., Category 7), if a much more severe event occurred in the future.

We readily acknowledge that we might still be missing events. However, given the number and type of sources used (which favour national-scale and/or especially damaging events), we are confident we have documented the most severe events that have occurred in the UK since 1915, although the severity of some events may have been under-ranked due to the lack of detailed information available. For a large number of Category 1 events, we were only able to identify the location(s) flooded and no specific consequences. These events are not likely to be Category 3 or higher, as from our experience the events of most extreme consequences were reported in more detail. Our approach is designed to allow improvement and if additional more detailed descriptions of flood impacts emerge, then we can re-rank these events accordingly.

We have not (due to time-constraints) directly considered wave data when compiling this database. In the event commentaries we referred to reported information on wave conditions, where available. In the future, we hope to add wave measurements from Centre for Environment, Fisheries and Aquaculture Science’s (CEFAS) Wavenet (https://www.cefas.co.uk/cefas-data-hub/wavenet/) and Coastal Channel Observatory (http://www.channelcoast.org) wave buoy datasets, for events, where the data is available. A limitation of wave datasets is their short duration over the last decade or two. An alternative would be to use multi-decadal model wave hindcasts.

We plan to update the database forwards in real-time, including any new coastal flood events as they happen. Just prior to submitting this paper, minor flooding (Category 2) occurred in Swanage and Portsmouth on the 19th November 2016, and we have added this event to the database, and in Whitby on 13th January 2017 (we haven’t added this event to the dataset as we wanted it to encompass complete years). In regard to the former event, we were able to visit both sites shortly after the event. We spoke to local coastal engineers and managers and obtained photos (and took our own) to build up an accurate picture of the extent and severity of the consequences that occurred, which helped to write the event commentary. We hope to be able to do this for future events. Also, we plan to continually update the database using new sources of information that reveal previously unidentified events, and expand upon the information available for events already within the updated record. By publishing this paper, making the dataset freely available via the SurgeWatch website and BODC portal, and regular publicity, we hope to promote and encourage identification of additional information regarding past events of which we are currently unaware. We actively encourage any interested parties such as coastal engineers, mangers or members of the public, to send us any information on flooding that is not yet featured in the database. All contributions that are included will be acknowledged.

Finally, we will shortly start to develop SurgeWatch3.0. We plan to repeat the tasks undertaken here, considering the same nine key sources, but for the pre-1915 events and go back in time as far as possible. In an initial assessment we have identified at least 400 events pre-1915, with the earliest event in 245 A.D. Going back pre-1915 we expect a large reduction in the amount of information available and increased uncertainty about the events. Extending the data back in time, we would also need to consider tsunamis.

## Additional Information

Tables 1 and 2 are only available in the online version of this paper.

**How to cite this article:** Haigh, I. D. *et al.* An improved database of coastal flooding in the United Kingdom from 1915 to 2016. *Sci. Data* 4:170100 doi: 10.1038/sdata.2017.100 (2017).

**Publisher’s note:** Springer Nature remains neutral with regard to jurisdictional claims in published maps and institutional affiliations.

## Supplementary Material



## Figures and Tables

**Table 1:** A summary of the characteristics of the nine main sources used to create the updated coastal flood database.

	**Source**	**Proxy for**	**Description**	**Number of coastal flood events**	**Coverage**	**Study area**	**Comment(s)**
1.	Lamb^35^	Journals, periodicals, archives	A record of historical North Sea storms, with varying details of meteorological conditions and other available information for each storm.	18	1509–1990	North Sea	The author acknowledges that the record cannot be complete, but is ‘most nearly’ complete with regard to the severest of storms in the region. With the focus on storms and the associated weather conditions, there is not much reference to floods and their details.
2.	Davison *et al.*^36^	Newspaper articles, books, archives, and other sources	A record of ‘dramatic weather events’ in Hampshire & the Isle of Wight.	3	1600–1993	Hampshire and the Isle of Wight	A record of extreme weather for the specified region, mainly with reference to weather-related phenomena other than extreme storms and coastal flooding (e.g., snow storms, heatwaves).
3.	Hickey^37^	62 sources including newspapers, literature, archives	Volume 2: The most comprehensive existing flood chronology for Scotland.	55	1100–1991	Scotland	Many sources provide general coverage over study area, although Northwest Scotland ‘has almost no data’. Hickey (1997) appraises the quality of the data, noting that since 1800 A.D. the recorded occurrence of every coastal flood is ‘classified as being reliable’ (based on devised method of assessing the reliability & authenticity of data sources).
		Journal papers and books (primarily Lamb 1991)	Volume 1: A record of floods in Northwest Europe (11 countries) excluding Scotland. Mainly relies on Lamb (1991) for England (east coast) floods during 19th century.	55	800–1990	NW Europe (exc. Scotland)	Mainly focused on major North Sea events, and the author notes that there is a ‘very detailed chronology for the east coast, [and] poor coverage for other [south & west] coasts’. A precise date is not always provided.
4.	Zong & Tooley^38^	*The Times Digital Archive* (primarily)	A review of microfilms of *The Times* newspaper & other archive sources.	134	1785–2003	UK	Coverage may be biased towards large-scale floods with national relevance, although records are accurate in terms of date and location (as opposed to water level, etc.). Online searches of the archive without use of a dedicated index not entirely reliable as digitised documents
5.	Eden^39^	Periodicals, personal notes, newspaper articles	A record of severe weather events (from snow/wind/hail storms, tornados, flash floods etc.).	35	1901–2008	UK	The author notes ‘no claim is made to have included every severe weather event, but serious omissions should be few’. Lacking detail in some cases with broad references to locations and date.
6.	Ruocco *et al.*^40^	Primarily two local newspapers: *The Echo* and *The News*	Review of local newspaper archives focusing on the Solent, south coast based on dates of the top 100 sea levels recorded by tide gauges in Southampton and Portsmouth.	69	1935–2005	Solent, south coast UK	Identifies definite occurrences of floods, with the dates deemed very reliable given that the sources used are local and contemporary. The record is however based on the dates of the highest sea levels, and is not an attempt to identify all coastal floods in the area, but presents a sufficiently reliable overview of at least more considerable events.
7.	Kundewicz *et al.*^41^	Newspaper articles, government reports, primary data	An ‘active archive of large flood events’ from around the world focusing on major events only.	4	1985–2010	Global	Approximately 50 floods for the UK in the database from 1989–2009, many of which are largely if not entirely fluvial/pluvial in origin, and none are classified as coastal floods, although our searches revealed that some events are known to have had marine influence.
8.	Stevens *et al.* (2014)	Met Office Monthly Weather Reports	Summary of the monthly weather published by the UK Meteorological Office.	43	1884–1993	UK	Consistent style of reporting through time, but floods tend to be mentioned briefly and not always attributed to a specific source. Locations often simplified to county or regional level, and exact dates are not always given.
		Monthly Hydrological Summaries (CEH)	Monthly UK hydrological summaries provided by CEH as part of the National Hydrological Monitoring Programme.	3	1988–present	UK	Purpose is not specific to coastal & marine hydrology, but there is occasionally a reference to coastal floods for more notable events. This record is useful because it extends over a period for which Met Office monthly reports are not available.
		UK Climate summaries (Met Office)	A general summary of the main weather features for each month published by the UK Meteorological Office.	6	2001–present	UK	Reports of flooding usually brief much like the discontinued reports from earlier, but this record is equally if not more reliable than earlier Met Office publications given that these reports are supported by widespread modern measurement instruments and real-time information.
9.	Haigh *et al.*^32^	Various sources including newspaper articles, periodicals, journal papers	Existing SurgeWatch1.0 database provides meteorological conditions, sea levels, and reports of coastal flooding for 96 high sea level events from 1915 to 2014.	59	1915–2014	UK	The events within the database are based on instances when observed sea level exceeded a 1 in 5 year return period, which provides an incomplete but useful record of events, particularly post-1980s when the national tide gauge coverage was comprehensive.

**Table 2:** A 102-year record of UK coastal flood events

**Event**	**Year**	**Month**	**Day**	**Category**	**County, region or country**	**Location(s)**	**References**
1	1915	9		2	North East Scotland (North Sea)	Nairn, Buckie	Hickey (1997)
2	1915	11		2	North East Scotland (North Sea)	Nairn	Hickey (1997)
3	1916	1	13–16	1	North Sea (England)	Essex, Kent, Humberside	Hickey (1997)
4	1916	9	14	1	North Sea (England)	Blakeney Point (North Norfolk)	Hickey (1997)
5	1916	11	5	3	English Channel (the Solent, Dorset)	Portsmouth, Bournemouth, Cowes	Zong & Tooley (2003)
6	1917	1	26	4	English Channel (South West)	Hallsands (Devon)	Zong & Tooley (2003)
7	1918	2	28–2	1	North Sea (England)	East Anglia, Thames Estuary, Kent	Eden (2008)
8	1918	1	15	2	English Channel (the Solent)	Shoreham, Littlehampton, Southampton	Zong & Tooley (2003); Eden (2008)
9	1919	2	17	1	North Sea (North East England)	Grimsby	Zong & Tooley (2003)
10	1920	1	8–9	2	North Sea (Scotland)	Perth (Tayside), Dundee	Hickey (1997); Zong & Tooley (2003)
11	1920	5	29	2	North Sea (England)	Louth, Grimsby	Zong & Tooley (2003)
12	1921	7	29	1	North Sea (South East England)	Folkestone	Zong & Tooley (2003)
13	1921	12	18	2	North Sea (Scotland)	Alloa	Hickey (1997)
14	1921	11	1	3	North Sea (England)	Thames Estuary, Kent, Lincolnshire, East Anglia, Scotland	Hickey (1997); Eden (2008); Zong & Tooley (2003)
15	1921	12	17	4	North Sea (North East England, Scotland)	Hull, Grimsby, Blyth, Teeside, Alloa, Dundee	Met Office (1921); Hickey (1997); Zong & Tooley (2003); Eden (2008)
16	1922	1	1	1	North Sea (South East England)	Southend	Hickey (1997)
17	1922	3	8	1	English Channel, North Sea (South East England)	Margate, Dover, Folkestone	Zong & Tooley (2003)
18	1922	4	14	1	Thames, Bristol Channel	London, Sharpness	Zong & Tooley (2003)
19	1922	10	11	1	North Sea (England)	Essex, Kent	Hickey (1997)
20	1922	10	27	1	North Sea (England)	Skegness (Lincolnshire)	Zong & Tooley (2003)
21	1923	2		2	Irish Sea (Scotland)	Dumfries	Hickey (1997)
22	1923	10	10–12	3	North Sea (England), English Channel (the Solent)	Hull, Scarborough, Severn Beach (near Bristol), Portsmouth, Hastings, Folkestone, Hythe, Sandgate, Dover (Kent)	Zong & Tooley (2003)
23	1924			1	Atlantic-Celtic Sea (England)	Isles of Scilly (Cornwall)	Hickey (1997)
24	1924	1		1	English Channel (Dorset)	Isle of Portland	West (2014)
25	1924	2		2	North Sea (North East England)	Blyth (Northumberland)	Northumberland County Council (2010)
26	1924	12	27	2	Irish Sea (England), North Sea (South East England), English Channel (the Solent)	Southsea (Portsmouth, Hampshire), Folkestone, Sandgate, Deal (Kent), Blackpool, Fleetwood, Lytham, Sandylands (Lancashire), Gretna, Dundee	Met Office (1924); Hickey (1997); Zong & Tooley (2003)
27	1925	2	21	1	Irish Sea (England)	Morecambe, Pilling, Knott End-on-Sea, Bolton-le-Sands, Cockerham Sands (Lancashire)	Zong & Tooley (2003)
28	1925	11	8	1	English Channel-North Sea (South East England)	Folkestone	Zong & Tooley (2003)
29	1925	11	25	1	North Sea (England)	—	Zong & Tooley (2003)
30	1926	2	19	1	Irish Sea (England)	Morecambe, Sandylands (Lancashire)	Zong & Tooley (2003)
31	1926	10	10	1	North Sea (England)	Humberside, Kent	Hickey (1997); Haigh *et al.* (2015)
32	1926	12	30	1	Irish Sea (North West England)	Preston, Heysham, Morecambe	Zong & Tooley (2003)
33	1926	11	4–5	2	Irish Sea-Atlantic-Firth of Clyde (West Scotland)	Broomielaw, Kirkcudbright, Glasgow	Met Office (1926); Hickey (1997); Zong & Tooley (2003); Eden (2008)
34	1927			1	Irish Sea (Wales, England)	North Wales, Lancashire	Hickey (1997)
35	1927	1	28	1	Irish Sea (England)	Fleetwood (Lancashire), Wales	Lamb (1991)
36	1927	2	5	1	North Sea (Thames)	Chiswick (Greater London)	Zong & Tooley (2003)
37	1927	3	5	1	North Sea (Thames)	Eel Pie Island (Greater London)	Zong & Tooley (2003)
38	1927	9	19	1	North Sea (England)	Boston (Lincolnshire)	Zong & Tooley (2003)
39	1927	9	23	1	North Sea (England)	Scarborough, Folkestone, Dover	Zong & Tooley (2003)
40	1927	11	9	1	North Sea (Thames)	London	Zong & Tooley (2003)
41	1927	12	26	1	North Sea (England), English Channel (Dorset)	Lowestoft, Thames Estuary, Herne Bay, Deal, Dover, Chesil Beach, Isle of Portland	Hickey (1997); Zong & Tooley (2003)
42	1927	10	28–29	5	Celtic Sea, Irish Sea	Mersey, Fleetwood, Blackpool, Sandylands, Cardigan Bay, Criccieth, Aberglaslyn, Porthmadog (Portmadoc)	Met Office (1927); Lamb (1991); Hickey (1997); Zong & Tooley (2003); Eden (2008)
43	1928	2	16	1	English Channel (the Solent)	Alum Bay (Isle of Wight)	Zong & Tooley (2003)
44	1928	10	28	1	Irish Sea (England)	Grange-over-Sands (Morecambe Bay, Cumbria)	Zong & Tooley (2003)
45	1928	3	22–23	2	North Sea (England)	Hull, Berwick-upon-Tweed	Zong & Tooley (2003); Haigh *et al.* (2015)
46	1928	1	6	5	North Sea (Scotland, Thames, South East England)	London (City), Southwark, Putney, Hammersmith, Westminister, Mersea, Maldon (Essex), Norfolk, Stranraer	Met Office (1928); Lamb (1991); Hickey (1997); Zong & Tooley (2003); Eden (2008)
47	1929	1	16	1	North Sea (South East England)	Southend	Hickey (1997)
48	1929	12	22	1	North Sea (South East England)	Southend	Hickey (1997)
49	1930	8		1	Irish Sea (South West Scotland)	Annan	Hickey (1997)
50	1930	8	14	1	Atlantic-Firth of Clyde (West Scotland)	Gourock, Lamlash (Isle of Arran)	Hickey (1997)
51	1930	9	24	1	North Sea (England), Irish Sea (Scotland)	Hull, Owston Ferry, Summergate Merse, Waterfoot, Hillend, Annan, Welldale (Dumfries)	Hickey (1997); Zong & Tooley (2003)
52	1930	11	8	1	North Sea (England)	London, Southend	Hickey (1997); Zong & Tooley (2003)
53	1930	2	1	2	English Channel (Dorset)	Isle of Portland, Chesil Beach (Dorset)	Zong & Tooley (2003)
54	1930	3	16	2	English Channel (East Sussex)	Winchelsea	Zong & Tooley (2003)
55	1931	3	6	1	Irish Sea (North Wales)	Rhos-on-Sea (Conwy County)	Zong & Tooley (2003); Haigh *et al.* (2015)
56	1931	12	28	1	North Sea (South East England)	Southend	Hickey (1997)
57	1931	8	18	2	Irish Sea (England), English Channel (Sussex)	Seaford, Fleetwood	Zong & Tooley (2003)
58	1931	11	10–11	3	English Channel (Solent, Sussex)	Winchelsea, Rye, Shoreham-by-Sea, Littlehampton, Freshwater Bay	Met Office (1931); Lamb (1991); Hickey (1997); Eden (2008)
59	1932	9	4	1	North Sea (South East England)	Southend	Hickey (1997)
60	1933	1	3	1	North Sea (England, Scotland)	Humberside, Highlands	Hickey (1997)
61	1933	10	11	1	North Sea (England, Scotland)	Humberside, Highlands	Hickey (1997)
62	1934	1	18	1	North Sea (South East England)	Southend	Hickey (1997)
63	1935	10	18–19	1	Atlantic (West Scotland)	Firth of Clyde	Zong & Tooley (2003)
64	1935	2	6	2	South East England (North Sea), South England (English Channel)	Southend, Benfleet (Essex), Cowes (Isle of Wight)	Hickey (1997); Zong & Tooley (2003)
65	1935	9	15–16	2	North Sea (England)	Barton-on-Humber (North Lincolnshire)	Hickey (1997); Zong & Tooley (2003)
66	1935	9	17	2	English Channel (Solent, Dorset)	Southampton, Milford, Netley, Keyhaven, Cowes, Bournemouth	Ruocco *et al.* (2011)
67	1936	2	23	1	North Sea (England, Scotland)	London, Kirkcaldy, Aberdeen	Zong & Tooley (2003)
68	1936	4	22	1	North Sea (South East England)	Southend	Hickey (1997)
69	1936	6	18	1	English Channel (the Solent)	Southsea (Portsmouth)	Zong & Tooley (2003)
70	1936	10	18	1	North Sea (South East England)	Southend	Lamb (1991); Hickey (1997)
71	1936	10	31	1	North Sea (South East England)	Southend	Hickey (1997)
72	1936	11	1	1	North Sea (Thames)	London	Zong & Tooley (2003)
73	1936	11	12	1	English Channel (Dorset), Irish Sea (Isle of Man)	Isle of Portland, Chesil Beach (Dorset), Castletown (Isle of Man)	Zong & Tooley (2003)
74	1936	12	14	1	Atlantic-Irish Sea (Scotland)	Ayr	Hickey (1997)
75	1936	12	18	1	Celtic Sea (Wales)	Cardiff	Zong & Tooley (2003)
76	1936	1	9–10	2	Celtic-Irish Sea, Atlantic (West Scotland)	Newport, Troon, Largs, Brodick, Rothesay, Ardrossan, Lamlash	Hickey (1997); Zong & Tooley (2003)
77	1936	3	1	2	North Sea (England)	Hull	Zong & Tooley (2003)
78	1936	12	30-1	3	North Sea (England)	London, Lowestoft, Great Yarmouth, Southend, Ramsgate	Hickey (1997); Zong & Tooley (2003)
79	1937	1	16–22	1	Atlantic-North Sea (Scotland)	Shetland	Hickey (1997)
80	1937	1		1	North Sea (Scotland)	Lossiemouth	Hickey (1997)
81	1937	11	20	1	North Sea (South East England)	Southend	Hickey (1997)
82	1937	1	27–28	2	Irish Sea (North Wales)	Rhos-on-sea, Beaumaris	Met Office (1937); Zong & Tooley (2003); Haigh *et al.* (2015)
83	1938	1	17	1	North Sea (South East England)	Southend (Essex)	Hickey (1997); Zong & Tooley (2003)
84	1938	1	29	1	Irish Sea (Wales, England), Bristol Channel	Bristol Channel, Cardigan Bay, Lancashire, Cumbria	Eden (2008)
85	1938	11		2	Irish Sea (Scotland)	Gretna, Rockcliff Marsh, Solway Firth	Hickey (1997)
86	1938	4	3	2	North Sea (England)	Horsey, London	Zong & Tooley (2003)
87	1938	1	15	3	Irish Sea (Wales, England), Bristol Channel	Bristol Channel, Cardigan Bay, Lancashire, Cumbria, Aberystwyth	Eden (2008)
88	1938	2	12–13	3	North Sea (England)	Yorkshire, Margate, Cromer, Maldon, London, Felixstowe, Grimsby	Met Office (1938); Lamb (1991); Hickey (1997); Zong & Tooley (2003); Eden (2008)
89	1939	1	20	1	English Channel (Dorset)	Isle of Portland	Zong & Tooley (2003)
90	1939	3	9	1	Irish Sea (England)	Fleetwood	Zong & Tooley (2003)
91	1942	2	13	1	English Channel (Dorset)	Isle of Portland	Zong & Tooley (2003)
92	1942	12	20	1	Irish Sea (Scotland)	Solway Firth	Zong & Tooley (2003)
93	1942	12	9–10	2	Irish Sea (Scotland-Solway Firth)	Solway Firth, Gretna, Annan	Hickey (1997)
94	1942	12	13	3	English Channel (Dorset), Irish Sea (Wales, Scotland)	Isle of Portland, Aberystwyth, Solway Firth	Met Office (1942)
95	1943	1	30	1	English Channel (Sussex)	Sussex	Zong & Tooley (2003)
96	1943	4	6	1	North Sea (South East England)	Southend	Zong & Tooley (2003)
97	1945	9	24	1	Irish Sea (Wales)	Rhyl (Denbighshire), Penmaenmawy, Llanfairfechan, Towyn (Conwy County)	Zong & Tooley (2003)
98	1945	12	21	1	English Channel (South West)	Starcross (Exe Estuary), Dawlish, Teignmouth (Devon)	Zong & Tooley (2003)
99	1945	12	24	1	English Channel (Sussex)	Seaford, Hastings	Zong & Tooley (2003)
100	1945	12	18–19	2	English Channel (Dorset, the Solent, Sussex)	Seaford, Isle of Portland, Hayling Island (Havant)	Zong & Tooley (2003); Ruocco *et al.* (2011)
101	1946	12	8	2	North Sea (England), English Channel (Sussex)	Lowestoft, Hastings, Pevensey Bay, Seaford, Eastbourne, Sandgate	Zong & Tooley (2003)
102	1947			1	North Sea (England)	The Fens	Hickey (1997)
103	1947	1	17	1	Irish Sea (England-Solway Firth-River Eden)	Carlisle	Zong & Tooley (2003)
104	1947	3	15–16	1	North Sea	—	Hickey (1997); Eden (2008)
105	1947	11	12	1	English Channel (Sussex)	Seaford	Zong & Tooley (2003)
106	1947	12	13	1	English Channel (Dorset)	Isle of Portland	Eden (2008)
107	1947	12	27	1	English Channel (the Solent, Sussex, Kent)	Emsworth, Hastings, Sandgate	Zong & Tooley (2003)
108	1947	1		2	North Sea (Scotland)	Kirkcaldy	Hickey (1997)
109	1947	4	21–23	2	Irish Sea (Scotland-Solway Firth-Rivers Annan and Nith)	Annan, Tay, Welldale (Dumfries)	Hickey (1997)
110	1948	1	29	2	English Channel (South West)	Looe, Saltash, Brixham	Zong & Tooley (2003); Haigh *et al.* (2015)
111	1948	8	8–11	1	North Sea (Scotland, England), English Channel (Kent-Sussex)	Berwick, Eyemouth, Jaywick, London, Felpham, Hastings, Folkestone, Sandgate	Zong & Tooley (2003)
112	1949			1	English Channel (Dorset)	Chesil Beach	West (2014)
113	1949	1	8	1	North Sea (England, Scotland)	Norfolk, Aberdeen, Dunbar	Hickey (1997)
114	1949	11	21	1	English Channel (Kent)	Folkestone, Sandgate	Zong & Tooley (2003)
115	1949	12		1	Scotland	—	Hickey (1997)
116	1949	10	23	2	English Channel (the Solent-Kent)	Hastings, Hythe, Folkestone, Sandgate, Southampton	Lamb (1991); Zong & Tooley (2003); Ruocco *et al.* (2011)
117	1949	3	1	4	North Sea (England), Thames	London, Sheerness, Margate, Southend, Woodbridge, Boston, Kings Lynn	Met Office (1949); Hickey (1997); Zong & Tooley (2003)
118	1950	2	3	1	English Channel (Dorset, the Solent)	Lymington, Keyhaven, Milford, Keyhaven, Warsash, Bournemouth	Ruocco *et al.* (2011)
119	1950	2	6	1	English Channel (the Solent)	Fawley (Hampshire)	Ruocco *et al.* (2011)
120	1950	2	16	2	Irish Sea-Atlantic-Firth of Clyde (West Scotland), North Sea-River Tay (East Scotland)	Aberfoyle, Perth, Helensburgh	Hickey (1997)
121	1951	12	30	1	Scotland	—	Lamb (1991)
122	1951	12	28–29	2	English Channel (Cornwall to Kent)	Southampton, Beaulieu, Shanklin, Sandown, Cornwall, Kent, St Leonards, Sussex	Lamb (1991); Zong & Tooley (2003); Ruocco *et al.* (2011)
123	1952	1	30	1	Celtic Sea (England)	Bude (North Cornwall)	Zong & Tooley (2003)
124	1952	8	10	1	English Channel (central)	Seaford	Zong & Tooley (2003)
125	1953	11	1	1	English Channel (east)	Sandgate (Kent)	Zong & Tooley (2003)
126	1953	9	23	2	Bristol Channel	Bristol, Pill-on-Avon	Zong & Tooley (2003); Haigh *et al.* (2015)
127	1953	1	31	6	North Sea (England), Thames	Kent, Spurn Head, Humber, London	Met Office (1953); Lamb (1991); Hickey (1997); Zong & Tooley (2003); Eden (2008); Haigh *et al.* (2015);
128	1954	1	3	1	North Sea (England), Irish Sea (England), Celtic Sea (North Devon)	Wells, Alderburgh, Barmston, Bude	Hickey (1997); Zong & Tooley (2003)
129	1954	9	1	1	North Sea (Thames)	Richmond on Thames (London)	Zong & Tooley (2003)
130	1954	10	6–7	1	North Sea (England)	—	Hickey (1997)
131	1954	11		1	North Sea (North East England)	Blyth	Northumberland County Council (2010)
132	1954	12	1	1	English Channel (Dorset)	Isle of Portland	West (2014)
133	1954	12	4–5	1	North Sea (England)	—	Hickey (1997)
134	1954	12	22	1	North Sea (England)	Great Yarmouth	Lamb (1991); Zong & Tooley (2003)
135	1954	9	14	2	Bristol Channel	Ashton Gate, Bristol	Zong & Tooley (2003)
136	1954	10	14	2	North Sea (North East England)	Hull	Zong & Tooley (2003)
137	1954	11	30	2	English Channel (Dorset, the Solent)	Christchurch, Lymington, Southampton	Ruocco *et al.* (2011)
138	1954	12	8	2	English Channel (the Solent)	Ryde, Lymington, Southampton	Ruocco *et al.* (2011)
139	1954	11	11	4	North Sea (England), English Channel (the Solent)	Hull, Southend, Strood (River Medway), Southampton	Met Office (1954); Zong & Tooley (2003); Ruocco *et al.* (2011)
140	1954	11	26	4	English Channel (Cornwall to Kent)	Lostwithiel, Gunnislake, Truro, Mevagissey, Perranporth, Chesil Beach, Isle of Portland, Worthing, Teignmouth, Newhaven, Seaford, Southampton, Bournemouth, Lymington, Isle of Wight	Met Office (1954); Zong & Tooley (2003); Ruocco *et al.* (2011)
141	1955	1	11	1	North Sea (England), Thames	Putney, Millbank (London), Tilbury, Southend (Essex), Hull, Scarborough, Cleethorpes (Yorkshire-Humber)	Zong & Tooley (2003)
142	1955	1	14	1	Bristol Channel-Celtic Sea (North Devon)	Weare Giffard (Devon)	Zong & Tooley (2003)
143	1955	2	24	1	North Sea (England)	Scarborough (North Yorkshire), Cleethorpes, Sandilands (Lincolnshire)	Zong & Tooley (2003)
144	1955	3	24	1	English Channel (Dorset, the Solent)	Christchurch, Southampton	Ruocco *et al.* (2011)
145	1955	10	6–7	1	North Sea (England)	—	Hickey (1997)
146	1955	10	15–17	1	North Sea (England)	—	Hickey (1997)
147	1955	12	8	1	North Sea (England)	—	Hickey (1997)
148	1956	1	17–21	1	North Sea (England)	—	Hickey (1997)
149	1956	3	2	1	North Sea (England)	—	Hickey (1997)
150	1956	1	30	2	East Scotland (North Sea, Firth of Forth)	Kirkcaldy, Wick	Hickey (1997)
151	1957	2	16	2	Celtic Sea (England), Bristol Channel, North Sea (Scotland)	Bridgewater, Combwich, Ilfracombe, Crovie, Gardenstown	Hickey (1997); Zong & Tooley (2003)
152	1957	9	24	2	Celtic Sea (England)	Westward Ho!, Bideford, Appledore, Instow, Ilfracombe	Zong & Tooley (2003)
153	1957	12	10	2	English Channel (Cornwall-Devon-Dorset)	Starcross, Saltash, Topsham, Weymouth, Bournemouth	Zong & Tooley (2003)
154	1958	10	15	1	North Sea (Thames)	Putney (London)	Zong & Tooley (2003)
155	1958	4	4	3	North Sea (Scotland)	Kirkcaldy, Edinburgh	Hickey (1997)
156	1959	10	17	2	English Channel (the Solent)	Yarmouth, Gurnard, Newport, Lymington	Ruocco *et al.* (2011)
157	1959	12	3	2	English Channel (the Solent)	Cowes, Hythe (Hampshire), Marchwood (Southampton)	Ruocco *et al.* (2011)
158	1959	12	30	2	North Sea (England, Scotland)	Hull, Ipswich, Dundee	Zong & Tooley (2003)
159	1960	9		1	English Channel (Cornwall)	St Austell River	Cornwall Council (2011)
160	1960	10	8	1	English Channel (South West and the Solent), Celtic Sea (Devon),	Exmouth, Torquay (South Devon), Brendon (North Devon), Southampton	Zong & Tooley (2003)
161	1960	1	6	2	North Sea (Scotland)	Alloa, Stirling	Hickey (1997)
162	1961	3	20–21	1	North Sea (England)	-	Hickey (1997); Haigh *et al.* (2015)
163	1961	7	4	1	North Sea (South East England)	Southend	Zong & Tooley (2003)
164	1961	10	24–25	2	English Channel (Dorset, the Solent), Atlantic West Scotland	Portsmouth (Old Portsmouth, Eastney), Fareham, Langstone, Hayling, Cowes, Newport, Ryde, Totton, Southampton, North Skye	Ruocco *et al.* (2011); Haigh *et al.* (2015)
165	1962			1	Atlantic-Celtic Sea (England)	Isles of Scilly	Hickey (1997)
166	1962	1	18	1	English Channel (Dorset)	Chesil Beach	West (2014)
167	1962	1	10	2	English Channel (the Solent)	Hythe, Bournemouth, Ryde	Ruocco *et al.* (2011)
168	1962	1	12	2	English Channel (the Solent)	Eastoke, Hayling, Lymington, Milford-on-Sea	Ruocco *et al.* (2011)
169	1962	3	7	4	English Channel (Cornwall), Atlantic-Celtic Sea (England)	Penzance, Newlyn, Tolcarne Beach, Isles of Scilly	Met Office (1962); Haigh *et al.* (2015)
170	1963	11	1–4	2	English Channel (the Solent, Sussex)	Havant (Hayling Island, Emsworth, Langstone), Fareham, Southampton, Warsash, Bognor	Ruocco *et al.* (2011)
171	1963	11	19	2	North Sea (Thames), English Channel (Kent-Sussex)	London, Hastings, Folkestone	Zong & Tooley (2003)
172	1965	1	20	2	North Sea (England), English Channel (Dorset)	Dymchurch, Hull, Bournemouth	Zong & Tooley (2003)
173	1966	4	11	1	North Sea (Scotland)	Aberdeen	Hickey (1997)
174	1966	10	15–16	2	English Channel (Dorset, the Solent)	Mudeford, Hythe, Southampton, Lymington	Ruocco *et al.* (2011)
175	1967	9	4	1	Irish Sea (England)	Blackpool	Lamb (1991); Hickey (1997)
176	1967	10	4	1	English Channel (the Solent)	Hythe, Cowes, Beaulieu	Ruocco *et al.* (2011)
177	1967	11		1	English Channel (South West)	Bude, Grogley (River Camel), Polmorla, Perranporth, Lostwithiel, Par	Cornwall Council (2011)
178	1967	1		2	Irish Sea (Scotland-Solway Firth), North Sea (Scotland-Moray Firth)	Carsethorn, Nigg Bay	Hickey (1997)
179	1967	11	2–4	2	English Channel (the Solent, Sussex)	Bognor, Ryde, Cowes, Hayling, Fareham	Ruocco *et al.* (2011)
180	1967	2	27–28	4	Irish Sea (Scotland-Solway Firth), North Sea (Scotland-Moray Firth)	Kippford, Carsethorn, Southerness, Rockcliffe, Kirkcudbright, Creetown, Carseluith, Garlieston, Isle of Whithorn, Port William, Drummore, Port Patrick; Annan, Kingholm Quay, Glencaple, Powfoot and Browhouses	Haigh *et al.* (2015)
181	1968	12	20–21	2	English Channel (the Solent)	Hythe, Lymington, Ryde, Cowes, Hythe, Newport, Yarmouth (Isle of Wight), Portsmouth (Eastney), Southampton, Netley, Beaulieu	Ruocco *et al.* (2011)
182	1969	1	17	1	English Channel (the Solent)	Southsea (Portsmouth), Hayling Island, Emsworth (Havant), Cowes, Yarmouth (Isle of Wight)	Ruocco *et al.* (2011)
183	1969	11	9	1	English Channel (the Solent)	Cowes, Chichester Harbour, Southampton	Ruocco *et al.* (2011)
184	1969	2	19	2	English Channel (South West), North Sea (England)	Teignmouth, Dawlish, Paignton	Met Office (1969); Hickey (1997); Eden (2008)
185	1969	3	18	2	North Sea (Scotland)	Kirkcaldy	Met Office (1969)
186	1969	11	12	2	English Channel (the Solent)	Fareham, Emsworth, Portchester, Cowes	Ruocco *et al.* (2011)
187	1969	9	28–29	4	North Sea (England, Scotland)	Hull, Great Yarmouth, Whalsay (Shetland Islands)	Met Office (1969); Hickey (1997); Zong & Tooley (2003); Eden (2008); Haigh *et al.* (2015)
188	1970			1	North Sea (England)	Kent, Humberside	Hickey (1997)
189	1970	2	1	1	Atlantic-Irish Sea-Firth of Clyde (Scotland)	Inverkip, Greenock	Hickey (1997)
190	1971	10	19	1	English Channel (Kent)	Sandgate	Zong & Tooley (2003)
191	1972	2	15	1	Irish Sea (Scotland)	Isle Of Whithorn	Hickey (1997)
192	1972	2	2	2	Irish Sea (Scotland)	Isle Of Whithorn	Hickey (1997)
193	1973	7	16	1	North Sea (England)	Grimsby, Cleethorpes	Zong & Tooley (2003)
194	1973	9		1	Atlantic-Irish Sea-Firth of Clyde (Scotland)	Gourock	Hickey (1997)
195	1974	11	15	1	English Channel (Cornwall, Dorset, Sussex)	St Ives (Cornwall), Preston, Weymouth (Dorset), Cuckmere valley (East Sussex)	Zong & Tooley (2003)
196	1974	1	11	2	Celtic Sea (Wales, Devon), Atlantic (North West Scotland)	Amroth, Pembrokeshire (South Wales), Barnstaple (North Devon), Ireland (Sligo, Waterford, Cork), Stornoway (Scotland)	Met Office (1974); Lamb (1991); Hickey (1997); Zong & Tooley (2003); Haigh *et al.* (2015)
197	1974	1	12	2	English Channel (the Solent)	Hayling Island, Ryde	Ruocco *et al.* (2011)
198	1974	2	9–11	3	English Channel (Cornwall to Kent), Bristol Channel	Severn Valley, Dawlish, St Blazey, Millbrook, Par, Lewes, Christchurch, Folkestone, Plymouth, Portsmouth, Southampton, Cowes, Hayling	Met Office (1974); Zong & Tooley (2003); Ruocco *et al.* (2011); Haigh *et al.* (2015)
199	1975	1	30	1	Atlantic (North West Scotland)	Stornoway	Hickey (1997); Haigh *et al.* (2015)
200	1975	3	20	1	North Sea (England)	Lowestoft	Zong & Tooley (2003)
201	1975	1	28	2	English Channel (the Solent)	Langstone, Cowes, Newport, Ryde, Wallington, Portsmouth	Ruocco *et al.* (2011)
202	1976	9	14	1	English Channel (Cornwall)	Polperro	Zong & Tooley (2003)
203	1976	10	23	1	English Channel (the Solent)	Hayling Island	Ruocco *et al.* (2011)
204	1976	10	14–15	2	English Channel (Devon, Dorset)	Torquay, Isle of Portland	Zong & Tooley (2003)
205	1976	1	2–3	4	Irish Sea (England), Atlantic (North West Scotland), North Sea (North East England)	Hull, Lowestoft, Fleetwood, North Uist	Lamb (1991); Hickey (1997); Zong & Tooley (2003); Eden (2008); Haigh *et al.* (2015)
206	1977	11	14	1	Irish Sea (England)	Morecambe, Pilling	Zong & Tooley (2003)
207	1977	11	11–12	5	Irish Sea (England)	Fleetwood, Morecambe, Pilling, Blackpool, Lytham	Met Office (1977); Zong & Tooley (2003); Eden (2008); Haigh *et al.* (2015)
208	1978			1	Irish Sea (Wales, England)	Lancashire	Hickey (1997)
209	1978	2	8	1	Bristol Channel	Severn at Gloucester	Zong & Tooley (2003)
210	1978	2	18–20	1	English Channel (Devon, Dorset)	Isle of Portland	Met Office (1978)
211	1978	12	23–24	1	North Sea (Scotland)	Kirkcaldy (Fife)	Hickey (1997)
212	1978	2	26	2	English Channel (the Solent)	Hayling, Portsmouth	Ruocco *et al.* (2011)
213	1978	11	15	2	English Channel (the Solent)	Hythe, Calshot	Ruocco *et al.* (2011)
214	1978	12	13	4	English Channel (Dorset)	Isle of Portland	Met Office (1978); Zong & Tooley (2003)
215	1978	1	11–12	5	North Sea (England, Scotland), English Channel (the Solent)	Grampian coastline, Wells-next-the-Sea, King’s Lynn, Cleethorpes, Wisbech, Sandilands, Mablethorpe, Trusthorpe, Ingoldmells, Walcott, Deal, Alnmouth, Amble Harbour, Berwick-upon-Tweed, Blyth, Hayling, Cowes, Bembridge	Met Office (1978); Lamb (1991); Hickey (1997); Zong & Tooley (2003); Eden (2008); Ruocco *et al.* (2011); Haigh *et al.* (2015)
216	1979	10		1	Celtic Sea (North Cornwall, England)	Wadebridge, Padstow	Cornwall Council (2011)
217	1979	11		1	English Channel (South West), Celtic Sea (England)	Par (South Cornwall), Portreath (North Cornwall)	Cornwall Council (2011)
218	1979	12	13	1	English Channel (Dorset)	Isle of Portland	Zong & Tooley (2003)
219	1979	11		2	Irish Sea (Scotland)	Carsethorn (Dumfries)	Hickey (1997)
220	1979	1	4–5	4	English Channel (Devon)	Torcross, Beesands	Met Office (1979); Zong & Tooley (2003)
221	1979	2	13	4	English Channel (Dorset)	Isle of Portland	Lamb (1991); Zong & Tooley (2003); Eden (2008)
222	1980	3	19	1	North Sea (England)	Grimsby	Zong & Tooley (2003)
223	1980	4	20	1	North Sea (England)	Felixstowe	Zong & Tooley (2003)
224	1980	10	24–25	1	North Sea-Moray Firth (Scotland), English Channel (the Solent)	Burghead Bay, Findhorn, Wootton Bridge (Isle of Wight)	Hickey (1997); Ruocco *et al.* (2011)
225	1980	1	21	2	English Channel (the Solent)	Gosport, Hayling	Ruocco *et al.* (2011)
226	1981	3		1	Celtic Sea (River Camel), English Channel (River Fowey)	Sladesbridge (North Cornwall), Fowey (South Cornwall)	Cornwall Council (2011)
227	1981	3	9	1	Celtic Sea (Wales), English Channel (Dorset)	Bridgend, Cardiff, Swanage, Bridport	Zong & Tooley (2003)
228	1981	10		1	English Channel (Cornwall), Irish Sea (Cornwall)	Fowey (River Fowey), Wadebridge (River Camel), Truro (Truro River)	Cornwall Council (2011)
229	1981	12	30	1	English Channel (the Solent), Bristol Channel	Hayling Island, Cowes, Mudeford Bay, Yarmouth, Weston super Mare, Burnham on Sea, Minehead, Clevedon, Porlock, Watchet, Bridgenorth, Hythe, Sandgate	Zong & Tooley (2003)
230	1981	12	13–14	5	English Channel (the Solent), Bristol Channel	Somerset (Burnham on Sea, Brean, Weston, Uphill, Sand Bay, Wick St Lawrence, Kingston Seamoor, Clevedon, Pawlett), Portsmouth, Hayling Island, Langstone, Fareham, Ryde, Cowes, Freshwater, Yarmouth, Southampton	Met Office (1981); Eden (2008); Ruocco *et al.* (2011); Haigh *et al.* (2015)
231	1982	6		1	English Channel-River Camel (Cornwall)	Padstow	Cornwall Council (2011)
232	1982	10	16	2	English Channel (the Solent)	Hayling Island	Ruocco *et al.* (2011)
233	1983	12	19–20	3	English Channel (Cornwall, the Solent)	Penzance, Fowey, Looe, Hythe, Hayling, Portsmouth, Lymington, Ryde, Cowes, Southampton	Met Office (1983); Eden (2008); Ruocco *et al.* (2011)
234	1983	2	1–2	4	North Sea (England, Scotland), Irish Sea (England), English Channel (the Solent)	Lowestoft, Great Yarmouth, Redcar, Morecambe, Filey, Scarborough, Mablethorpe, Lossiemouth, Findhorn, Buckie, Portgordon, Kingston, Garmouth, Southampton, Cowes, Bembridge	Met Office (1983); Lamb (1991); Hickey (1997); Zong & Tooley (2003); Eden (2008); Ruocco *et al.* (2011); Haigh *et al.* (2015)
235	1984	4	16	1	Bristol Channel	Burnham on Sea	Zong & Tooley (2003)
236	1984	9	26	1	North Sea (Thames), English Channel (the Solent)	Putney, Ryde, Cowes	Zong & Tooley (2003); Ruocco *et al.* (2011); Haigh *et al.* (2015)
237	1984	10	24–25	2	Celtic Sea-River Camel (North Cornwall, England); English Channel (Dorset, the Solent)	Polmorla, Wadebridge, Padstow, Chapel Amble, Warsash (River Hamble), Fareham, Cowes	Davison *et al.* (1993); Ruocco *et al.* (2011)
238	1984	11	23–24	3	Celtic Sea-River Camel (North Cornwall, England); English Channel (the Solent, Sussex)	Fowey, Padstow, Wadebridge, Sladesbridge, Perranporth; Lymington, Portsmouth, Southampton, Cowes, Gurnard, Ryde, Fareham, Hythe, Hayling Island, Langstone, Shoreham	Met Office (1984); Ruocco *et al.* (2011); Haigh *et al.* (2015)
239	1985	1		1	English Channel-Carrick Roads (South West England)	Falmouth, Penryn	Cornwall Council (2011)
240	1985	11	9	1	North Sea (Scotland)	Buckie	Hickey (1997)
241	1985	12	26	1	Celtic Sea-Bristol Channel	Avon Valley	Zong & Tooley (2003)
242	1985	1	22	2	North Sea (Scotland)	Portgordon, Portessie, Buckie Loch, Shelly Head	Hickey (1997)
243	1985	4	6–8	3	English Channel (Cornwall, the Solent, Sussex), Celtic Sea (North Cornwall)	Portsmouth, Hayling Island, Eastney and Elmer (near Bognor Regis), Portchester, Hythe, Wadebridge, Padstow, Newquay, Hayle, Mousehole, Flushing, Mevagissey, St Blazey, Fowey, Lostwithiel, Looe, Torpoint, Calstock, Dartmouth	Zong & Tooley (2003); Ruocco *et al.* (2011); Haigh *et al.* (2015)
244	1986	11		1	English Channel-River Fowey (Cornwall)	Lerryn	Cornwall Council (2011)
245	1986	11	21	2	English Channel (South West and central), Celtic Sea (Wales)	Cornwall, Isle of Wight-the Solent (Cowes, Portsmouth), East Sussex, Wales	Met Office (1986)
246	1987	10	7–9	2	Celtic Sea (North Cornwall, England); English Channel (the Solent)	Boscastle, Chapel Amble, Polmorla (Wadebridge, River Camel), Lymington, River Hamble, Southampton, Cowes, Shanklin, Ryde, Fareham	Met Office (1987); Zong & Tooley (2003); Ruocco *et al.* (2011); Haigh *et al.* (2015)
247	1988	1		1	English Channel (Cornwall)	Lerryn, Lostwithiel (River Fowey), Looe	Cornwall Council (2011)
248	1988	1	5	1	English Channel (the Solent)	Ryde	Zong & Tooley (2003)
249	1988	3	2–4	1	North Sea (England)	Great Yarmouth, Burl Valley, Thurne Valley, Spurn Head	Zong & Tooley (2003); Lamb (1991); Hickey (1997)
250	1989	2	11	1	Atlantic (North West Scotland)	Stornoway	Hickey (1997)
251	1989	2	14	1	North Sea (England)	Brightlingsea, Wivenhoe, Southwold	Lamb (1991); Zong & Tooley (2003); Haigh *et al.* (2015)
252	1989	3	8	1	Irish Sea (Scotland)	Carsethorn (Dumfries)	Hickey (1997)
253	1989	12	20	1	English Channel (Devon, the Solent)	Sidmouth, Dawlish, Budleigh Salterton, Lympstone, Southampton	Zong & Tooley (2003)
254	1989	12	13–17	4	Atlantic-Celtic Sea (England); English Channel (the Solent)	Isles of Scilly, Southampton, Lymington, Gurnard, Gosport, Newport, Cowes, Fareham, Emsworth, Old Portsmouth, Selsey, Brockenhurst, Seaview, Hythe, Warsash, Isle of Portland, Keyhaven	Met Office (1989); Davison *et al.* (1993); Hickey (1997); Eden (2008); Ruocco *et al* (2011); Haigh *et al.* (2015)
255	1990	2		1	Celtic Sea (North Cornwall, England)	Newquay, St Ives	Cornwall Council (2011)
256	1990	10	28–30	1	English Channel	-	Eden (2008); Kundewicz *et al.* (2013)
257	1990	12	12	1	North Sea (England)	Norfolk, East Anglia	Hickey (1997)
258	1990	2	1–3	2	Irish Sea (Wales); Atlantic (North West Scotland)	Towyn, Stornoway; England (unknown locations)	Lamb (1991); Hickey (1993); Zong & Tooley (2003)
259	1990	2	26–27	5	Irish Sea (Wales)	Pensarn to Kinmel Bay, Towyn, Rhyl, Ffynnongroyw, Prestatyn, Clwyd	Met Office (1990); Lamb (1991); Kundewicz *et al.* (2013); Haigh *et al.* (2015)
260	1991	1	2	1	Irish Sea (England and North Wales)	Morecambe, Rampside, Barrow-in-Furness, Holyhead	Zong & Tooley (2003); Haigh *et al.* (2015)
261	1991	11		1	Irish Sea-Firth of Clyde (Scotland)	Ardrossan	Hickey (1997)
262	1991	1	5	4	Irish Sea-Firth of Clyde (Scotland)	Ardrossan, Helensburgh, Millport, Largs, Saltcoats, Rothesay	Met Office (1991); Hickey (1997); Kundewicz *et al.* (2013); Haigh *et al.* (2015)
263	1992	8	29	1	English Channel (Cornwall, Dorset)	Christchurch, Wadebridge, Hayle and Lelant, Newlyn, Penzance and Long Rock, Helston, Falmouth and Penryn, Perranarworthal, Truro, Pentewan, Bugle, St Blazey and Tywardreath, the Glyn Valley, East Taphouse	Met Office (1992); Haigh *et al.* (2015)
264	1993	11	14	1	North Sea (England)	Goxhill, Humberston, Ashby cum Fenby, Cayton near Scarborough	Zong & Tooley (2003); Haigh *et al.* (2015)
265	1993	1	10–13	3	English Channel (the Solent)	Portsmouth, Hayling, Fareham (Warsash, Portchester, Wallington), Gosport, Isle of Wight (Cowes, Ryde, Wootton Bridge, Newport), Southampton, Romsey	Davison *et al.* (1993); Met Office (1993); Eden (2008); Ruocco *et al.* (2011); Haigh *et al.* (2015)
266	1993	2	21	4	North Sea (England)	Humberside, Thames Estuary, Great Yarmouth, Spurn Head, Scarborough	Met Office (1993); Zong & Tooley (2003); Eden (2008); Haigh *et al.* (2015)
267	1994	12	4	1	Bristol Channel, English Channel (the Solent)	Severn valley at Gloucester, Portsmouth, Cowes, Hamble	Zong & Tooley (2003); Ruocco *et al.* (2011); Haigh *et al.* (2015)
268	1994	12	7	2	English Channel (the Solent)	Langstone, Gosport, Emsworth, Hayling, Botley	Ruocco *et al.* (2011); Haigh *et al.* (2015)
269	1995	12	23–24	1	English Channel (the Solent)	Portsmouth, Wootton Bridge, Hythe, Dibden	Ruocco *et al.* (2011); Haigh *et al.* (2015)
270	1995			2	North Sea (England)	Blyth	Northumberland County Council (2015)
271	1995	1	19	2	English Channel (the Solent)	Langstone, Gosport, Portsmouth, Southsea, Hayling, Southampton	Ruocco *et al.* (2011)
272	1996			1	North Sea (North East England)	Beadnell (Northunmberland)	Northumberland County Council (2015)
273	1996	2	19–20	1	North Sea (England)	East Yorkshire, Lincolnshire, Norfolk, East Kent	Eden (2008)
274	1996	10	28–29	1	English Channel (the Solent); Bristol Channel	Langstone, Emsworth, Gosport; Porlock Bay	Ruocco *et al.* (2011); Haigh *et al.* (2015)
275	1996	1		2	English Channel (Cornwall), Celtic Sea (North Cornwall)	Sladesbridge, Gillan Harbour, Lerryn, Polperro	Cornwall Council (2011)
276	1996	4		2	Celtic Sea-River Camel (North Cornwall, England)	Wadebridge	Cornwall Council (2011)
277	1997	2	10	1	Irish Sea (England)	Sefton coast	Haigh *et al.* (2015)
278	1997	2	24	1	English Channel (Southern England)	-	Eden (2008)
279	1997	12		1	English Channel and west coasts	-	CEH (1997)
280	1997	2		2	North Sea (North East England)	Blyth, Berwick-upon-Tweed, Holy Island	Northumberland County Council (2015)
281	1998	1	1–8	1	English Channel	-	Eden (2008)
282	1998	3	4	1	English Channel (the Solent)	Old Portsmouth	Ruocco *et al.* (2011)
283	1998	9	9	1	English Channel (the Solent)	Selsey, Langstone, Emsworth	Ruocco *et al.* (2011)
284	1998	1	14	2	English Channel (the Solent)	Selsey, Ryde	Ruocco *et al.* (2011)
285	1998	1	4	3	English Channel (the Solent)	Southsea, Selsey, Hayling, Gosport, Fareham	Ruocco *et al.* (2011)
286	1999	1	4	1	English Channel (the Solent)	Selsey	CEH (1999); Haigh *et al.* (2015)
287	1999	8		1	North Sea (North East England)	Northumberland	Northumberland County Council (2015)
288	1999	10	24	2	English Channel (the Solent)	Selsey, Southsea (Portsmouth)	Ruocco *et al.* (2011)
289	1999	12	24–25	3	English Channel (Dorset, the Solent, Channel Islands, Kent)	Portsmouth, Southampton, Selsey, Jersey, Lymington, Dorset, Kent	Eden (2008); Ruocco *et al.* (2011); Kundewicz *et al.* (2013); Haigh *et al.* (2015)
290	2000	10		1	UK	-	CEH (2000)
291	2000	12	12	2	English Channel (Dorset)	Christchurch	Haigh *et al.* (2015)
292	2001	3	10–12	1	English Channel (the Solent)	Emsworth	Ruocco *et al.* (2011); Haigh *et al.* (2015)
293	2002	1	29	1	English Channel (the Solent)	Hayling	Ruocco *et al.* (2011)
294	2002	9		2	North Sea (North East England)	Seahouses (Northumberland)	Northumberland County Council (2015)
295	2002	2	1	4	Celtic Sea, Irish Sea, English Channel	Barrow-in-Furness (Cumbria), Langstone, Southsea/Portsmouth, Hayling Island (Hampshire), Sladebridge (north Cornwall), Mevagissey, Polkerris, Fowey, Golant, Lerryn and Lostwithiel, Cremyll and Calstock (south Cornwall)	Eden (2008); Ruocco *et al.* (2011); Haigh *et al.* (2015)
296	2004	10	27	3	English Channel (Cornwall-Devon-Dorset)	Cornwall, Devon, Dorset	Met Office (2004); Eden (2008); Haigh *et al.* (2015)
297	2005	11	3	3	English Channel (Dorset, the Solent)	Chesil, Hayling Island	Ruocco *et al.* (2011)
298	2005	1	11–12	5	Atlantic (North West Scotland); North Sea (North East)	South Uist, Barra (Scotland), Warkworth (River Coquet, Northumberland)	Haigh *et al.* (2015)
299	2006	3		2	English Channel (Cornwall), Celtic Sea (North Cornwall)	Bude, Boscastle, Wadebridge, Perranporth, Portreath, Hayle, St Erth and Lelant, Newlyn, Penzance, Gweek, Flushing, Pentewan, Par, Fowey, Looe, Saltash	Cornwall Council (2011)
300	2006	3	30	2	Bristol Channel	Tintern	Haigh *et al.* (2015)
301	2006	10	6–8	2	Celtic Sea (Devon, Cornwall), English Channel (South West)	Widemouth Bay, Trebarwith Strand, Port Isaac, Polzeath, Mawgan Porth, Newquay, Perranporth, Portreath, St Ives, Penzance, Flushing, Penryn, Perranarworthal, Mevagissey, Fowey and Looe	Haigh *et al.* (2015)
302	2006	12	3	2	Celtic-Irish Seas, English Channel (South West England, Northern Ireland)	Devon, Cornwall	Haigh *et al.* (2015)
303	2007	2		1	North Sea (North East England)	Berwick-upon-Tweed	Northumberland County Council (2015)
304	2007	11	9	3	North Sea (England)	Walcott (Norfolk), Suffolk	Eden (2008); Haigh *et al.* (2015)
305	2008	3	10	3	English Channel (Cornwall, the Solent, Channel Isles)	Teignmouth, Flushing, Poole, Beaulieu, Totton, Southampton (St Denys, Woodmill), Portsmouth, Sandown, Cowes, Gurnard, Yarmouth (Isle of Wight), Bosham, Emsworth, Selsey, Jersey	Wadey *et al* (2013); Haigh *et al.* (2015)
306	2010	11	11	1	UK west and south	Wales, Western Scotland, Isle of Wight	Haigh *et al.* (2015)
307	2010	3	29–31	3	North Wales (Irish Sea), Scotland	Llanddulas (Conwy County), Rhyl (Denbighshire), Bangor (Gwynedd), St Andrews Golf Course (Fife), Edinburgh, East Lothian	Met Office (2010)
308	2011	11	27	2	North Sea (England)	Whitby, Scarborough, Humber Estuary, Norfolk	Haigh *et al.* (2015)
309	2012	10	17	1	English Channel (Dorset)	Studland, Poole (Dorset)	Haigh *et al.* (2015)
310	2012	10	18	2	English Channel (Cornwall)	Lynmouth, Mevagissey, Looe, Kingsbridge	Met Office (2012)
311	2012	12	14	3	English Channel (Cornwall), North Sea-Moray Firth (Scotland)	Looe, Lossiemouth	Met Office (2012)
312	2013	10	28	1	English Channel (the Solent)	Yarmouth (Isle of Wight)	IOWCP (2013a); IOWCP (2013b); Wadey *et al.* (2015); Ozsoy *et al.* (2016)
313	2013	12	6	5	North Sea (England, Scotland), English Channel (Kent to the Solent), Irish Sea (North Wales, England, Scotland), Atlantic Scotland	Sunderland, Hull, Boston, Great Yarmouth, Lowestoft, North Berwick, Jaywick, Blackpool, Cleveleys, Walcott, Cromer, Whitstable, Portgordon, New Brighton, Rhyl, Havant, Cowes, Southampton	Met Office (2013); Haigh *et al.* (2015)
314	2014	8	12	1	Irish Sea (England)	Overton, Sunderland Point (Lancashire)	BBC (2014)
315	2014	1	6	3	English Channel (Dorset), Celtic & Irish Seas (Wales)	Aberystwyth, Christchurch	Haigh *et al.* (2015)
316	2014	2	1	3	English Channel (South West), Celtic & Irish Seas (Wales)	Aberystwyth, Newgale, Looe, Newlyn	Haigh *et al.* (2015)
317	2014	2	3	3	English Channel (South West)	Looe, Fowey, Newlyn, Porthleven, Mevagissey, Plymouth, Salcombe, Exmouth, Kingsbridge (Estuary)	Haigh *et al.* (2015)
318	2014	2	14	3	English Channel (Devon, Dorset, the Solent)	Plymouth, Milford on Sea, Chesil Beach, Hurst Spit	Met Office (2014); Haigh *et al.* (2015)
319	2014	1	3	4	English Channel (Cornwall to Sussex, Channel Isles), Bristol Channel, Irish Sea (Isle of Man)	Isle of Man, Newquay, Minsterworth, Maisemore, Elmore, Newnham, Jersey, Hastings	Haigh *et al.* (2015)
320	2015	1	22	1	Irish Sea (England)	Lancaster	Lancaster Guardian (2015)
321	2015	2	20–21	1	North Sea (Thames)	Richmond on Thames (London)	De Peyer (2015); Mann (2015); ITV (2015)
322	2015	3	21	1	North Sea (Thames)	Greenwich (London)	The Independent (2015)
323	2015	8	4	1	Irish Sea (Northern Ireland)	Salthill (Galway)	BBC (2015)
324	2015	10	28	1	English Channel (Dorset)	Christchurch (Dorset)	SurgeWatch Blog
325	2015	12	30	2	Irish Sea-Firth of Clyde (Scotland)	Saltcoats	BBC (2016a)
326	2016	2	8	1	English Channel (Dorset, the Solent), Celtic Sea (Wales)	Poole, Portsmouth, Porthcawl, Aberywystwyth	BBC (2016b)
327	2016	4	10	2	English Channel (Cornwall)	St Maws, Porthleven, Looe, Cawsand	BBC (2016c)
328	2016	5	6	1	Celtic Sea-Bristol Channel (Wales)	Ogmore	Houghton (2016)
329	2016	11	19	2	English Channel (Dorset, the Solent)		

**Table 3:** Categories and associated criteria used to rank the historical flood events based on the severity of their consequences.

**Category**	**Description**	**Criteria**
6	**Disastrous floods**	***Events must meet the following two criteria plus ≥2 criteria from Category 5***
		Multiple structural breaches of defences
		≥1 fatality caused by drowning in ≥1 locations
5	**Severe floods:** severely threatens life and/or results in considerable damage to infrastructure and property	***Events must: (1) meet ≥1 of the following criteria, or be associated with ≥1 fatality during the event or aftermath, and (2) meet ALL Category 2 criteria AND ≥1 criteria from Category 4***
		Substantial institutional response during or after the event (e.g., emergency Cabinet Office meeting, all-day media coverage)
		Reliable evidence that multiple fatalities were prevented by defences and/or flood warnings
4	**Major floods:** Impacts reported in monetary terms but no known loss of life	***Events must meet ALL of the Category 2 criteria, and 1 of the following***
		Descriptions of deep and/or fast-flowing water in ≥1 location, and/or statements to the effect of the ‘worst flooding in [x number of] years’, or the ‘worst flooding since [reference event]’
		Serious damage to residential properties (e.g., loss of contents), and/or residential properties made uninhabitable due to coastal erosion
		Economic damages reported
		Damage to major infrastructure (e.g., port, key railway line, power station, motorway)
3	**Moderate floods:** Multiple impacts across affected areas (can be minor to quite severe)	***Events must meet ≥3 of Category 2 criteria***
2	**Minor floods:** Impacts known of, although full consequences may still be unclear	***Events must contain <3 of these criteria, or otherwise reports of several properties flooded***
		Any reference to flooded property or agricultural land and livestock
		Disruption to services (e.g., branch railway line, power substation), including transport networks
		Descriptions of inundation including words such as ‘extensive’, ‘deep’, ‘serious’, ‘widespread’, ‘cut off’, ‘severe’
		Damage to sea defences beyond limited overtopping
1	**Nuisance floods:** localised or unknown due to lack of information	Terms appear (with reference to flooding) in reports including: ‘localised’ or explicit statements that the impacts were limited e.g., few local roads, quayside areas
		No reports of flooded properties
		Insufficient information available to determine severity of consequences
